# The Protease Inhibitor Alpha-2-Macroglobuline-Like-1 Is the p170 Antigen Recognized by Paraneoplastic Pemphigus Autoantibodies in Human

**DOI:** 10.1371/journal.pone.0012250

**Published:** 2010-08-18

**Authors:** Isabelle Schepens, Fabienne Jaunin, Nadja Begre, Ursula Läderach, Katrin Marcus, Takashi Hashimoto, Bertrand Favre, Luca Borradori

**Affiliations:** 1 Department of Dermatology, Inselspital, Bern University Hospital and University of Bern, Bern, Switzerland; 2 Department of Dermatology, Geneva University Hospital, Geneva, Switzerland; 3 Medical Proteome Center (MPC), Ruhr University of Bochum, Bochum, Germany; 4 Department of Dermatology, Kurume University School of Medicine, Kurume, Japan; The University of Queensland, Australia

## Abstract

**Background:**

Paraneoplastic pemphigus (PNP) is a devastating autoimmune blistering disease, involving mucocutaneous and internal organs, and associated with underlying neoplasms. PNP is characterized by the production of autoantibodies targeting proteins of the plakin and cadherin families involved in maintenance of cell architecture and tissue cohesion. Nevertheless, the identity of an antigen of Mr 170,000 (p170), thought to be critical in PNP pathogenesis, has remained unknown.

**Methodology/Principal Findings:**

Using an immunoprecipitation and mass spectrometry based approach, we identified p170 as alpha-2-macroglobuline-like-1, a broad range protease inhibitor expressed in stratified epithelia and other tissues damaged in the PNP disease course. We demonstrate that 10 PNP sera recognize alpha-2-macroglobuline-like-1 (A2ML1), while none of the control sera obtained from patients with bullous pemphigoid, pemphigus vulgaris, pemphigus foliaceus and normal subjects does.

**Conclusions/Significance:**

Our study unravels a broad range protease inhibitor as a new class of target antigens in a paraneoplastic autoimmune multiorgan syndrome and opens a new challenging investigation avenue for a better understanding of PNP pathogenesis.

## Introduction

Paraneoplatic pemphigus (PNP) is an autoimmune multiorgan syndrome associated with an underlying neoplasia [1], [2], [3]. Associated neoplasms include non-Hodgkin's lymphoma, chronic lymphatic leukaemia, Castleman disease, thymoma, and poorly differentiated sarcomas. PNP patients characteristically develop a severe polymorphous mucocutaneous eruption, features of which resemble pemphigus vulgaris, erythema multiforme, Stevens-Johnson syndrome and/or lichen. Involvement of internal organs, such as pulmonary and gastrointestinal tracts, is also observed [2], [3], [4]. Histologically, skin and mucosal lesions typically present intraepithelial cleavage, suprabasal acantholysis, and interface changes with necrotic and apoptotic keratinocytes [1], [2], [3], [5], [6]. Direct immunofluorescence (IF) microscopy studies disclose intraepidermal and/or basement membrane zone deposition of IgG and/or C3 complement component, whereas by indirect IF microscopy PNP sera contain autoantibodies binding to stratified, complex and simple epithelia, as well as to the myocardium [1], [2], [3]. PNP patients' autoantibodies typically bind to variable proteins including the plakin family members desmoplakin I and II, envoplakin, periplakin, plectin, and the bullous pemphigoid antigen 230 (BP230, also termed BPAG1-e). Furthermore, in analogy to pemphigus, desmoglein (Dsg) 1 and Dsg 3 are also consistently recognized [1], [7], [8], [9]. Finally, PNP autoantibodies immunoprecipitate an unidentified protein of Mr170,000 (p170) from keratinocyte extracts [1], [7], [8], [9], [10], [11].

The pathological mechanisms underlying tissue damage in PNP remain unclear. Different pathways have been involved to explain the polymorphous clinical features and multiorgan involvement [3]. Ample evidence indicates that autoantibodies against the desmosomal components Dsg 1 and Dsg3, which are expressed in stratified epithelia, play a central role in disrupting cell-cell adhesion of keratinocytes leading to acantholysis and intraepidermal blistering [9]. The vast majority of PNP sera contain autoantibodies directed against members of the plakin family. These proteins serve as versative cytolinkers connecting the intermediate filament cytoskeleton to distinct membrane sites, such as desmososomes and hemidesmosomes. However, despite their ubiquitous expression, their role in disease initiation is unlikely, since they are cytoplasmic proteins [8], [11]. Furthermore, though the majority of PNP sera also immunoprecipitate the p170 autoantigen, the search for the identity of this protein has proved to be technically challenging [9], [12], [13]. Finally, CD8+ cytotoxic T lymphocytes and other mononuclear cells are likely to contribute to tissue damage resulting in keratinocyte necrosis and apoptosis with a graft-versus-host disease-like phenotype in the skin [6], [14].

Since identification of the target antigens is critical for a better understanding of the pathophysiology of a devastating multiorgan autoimmune syndrome such as PNP, we sought to characterize p170 by using a combination of immunoprecipitation and mass spectrometry analyses. We have identified A2ML1, a broad range protease inhibitor expressed in the epidermis and other tissues [15], as a novel autoantigen targeted by PNP autoantibodies.

## Results

### Analysis by MALDI-MS of p170 immunoprecipitated by a PNP serum

We first performed a preparative immunoprecipitation of PNP antigens from unlabelled cultured primary keratinocytes, differentiated for 5 days using a previously well characterized PNP serum sample [10]. Immunoprecipitated proteins were separated by 1D-SDS-PAGE and stained by Coomassie blue. The stained protein band migrating at Mr 170,000 was excised and subjected to MALDI-MS analysis. Mass profiles of the tryptic peptides are shown in **Figure S1** and the mono-isotopic masses derived from these profiles were used for the search in databases. We analyzed the data using the Mascot program (http://matrixscience.com) [16] to match the peptide mass fingerprint to two databases, NCBI and MSDB. When blasted against the NCBI database, the top score protein was alpha-2-macroglobuline-like-1 (A2ML1) (homo sapiens), with a calculated molecular mass of 161 kDa (gi|74271845, A8K2U0, Genbank accession No: AL832139). A total of 43 out of 128 peptides from trypsinized p170 matched the theoretical mass values of A2ML1 tryptic peptides. The 43 p170-tryptic peptides covered 43% of the A2ML1 sequence with an equal repartition of matches along the entire polypeptide. When blasted against the MSDB database, the top score was obtained for CAD48670, a protein of 165 kDa (sequence 1, homo sapiens, covered by patent WO0229058). There were 44 p170-tryptic peptides matching the theoretical peptide mass of trypsinized CAD48670, covering 43% of the whole sequence. CAD48670 represents a putative splice variant of A2ML1. Finding A2ML1 (A8K2U0 and CAD48670) in two different databases prompted us to hypothesize that p170 could be A2ML1.

### Screening of PNP serum samples positive for p170

**Figure 1:**
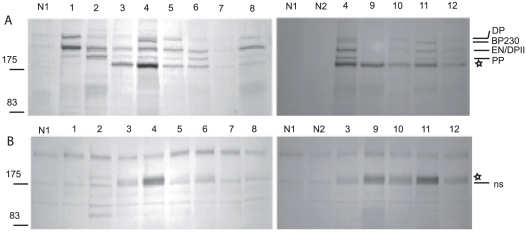
Analysis of keratinocyte proteins immunoprecipitated by PNP auto-antibodies. Normal sera (N1 and N2) or PNP sera (1–12) were used to immunoprecipitate proteins from radiolabelled keratinocyte extracts (A) or culture medium (B). Immunoprecipitates were denatured under reducing conditions, separated on 6% SDS-PAGE, and autoradiographed. p170 migration level is indicated by a star. PNP sera 1 and 2 immunoprecipitated negligible amounts of p170. BP230, desmoplakin (DPI, DPII), envoplakin (EN) and periplakin (PP) migration levels are indicated in panel A. In panel B, the non specific band also recognized by the control sera (N1, N2) with a slightly faster electrophoretic migration than p170 is indicated (ns).

As the MALDI-MS results were obtained from p170 immunoprecipiated by a single PNP serum, which was consumed for the preparative immunoprecipitation, we first tested additional PNP serum samples to further characterize p170. Sera obtained from 20 PNP patients were screened by conventional immunoprecipitation using biosynthetically radiolabeled extracts from cultured differentiated human keratinocytes. Analysis of reduced immunoprecipitation samples showed that most PNP sera targeted proteins of Mr 250,000, 230,000, 210,000, and 190,000 corresponding to desmoplakin I, BPAG1-e/BP230, desmoplakin II and/or envoplakin, and periplakin, respectively as described [3]. Furthermore, 10 out of the 20 PNP serum samples also immunoprecipitated p170 (**Figure 1A** **, serum 3 to 12**). In our experiments, p170 displayed a Mr slightly higher than previously observed, above the 175,000 marker. This is most likely due to the use of different, pre-stained molecular weight markers. The stoichiometry of the precipitated proteins varied among the various PNP sera. In contrast, two serum samples obtained from normal volunteers (**Figure 1A** serum N1 and N2) did not significantly immunoprecipitate any proteins.

### p170 is immunoprecipitated by PNP sera from the culture medium of keratinocytes

Since A2ML1 is a secreted protein [15], we next assessed whether p170 could be detected by immunoprecipitation with PNP sera from the culture medium of radiolabeled differentiated keratinocytes. Analysis of reduced immunoprecipitation samples showed that PNP sera reactive against p170 from keratinocyte extracts (sera 3 to 12) also recognized a 170,000 protein from the culture medium of keratinocytes (**Figure 1B**). The relative intensity of the bands recognized by the various PNP sera from culture media corresponded to that observed using keratinocyte extracts. These results indicate that p170, like A2ML1, is a secreted protein.

### Recognition of p170 by PNP sera is sensitive to reducing agents

**Figure 2:**
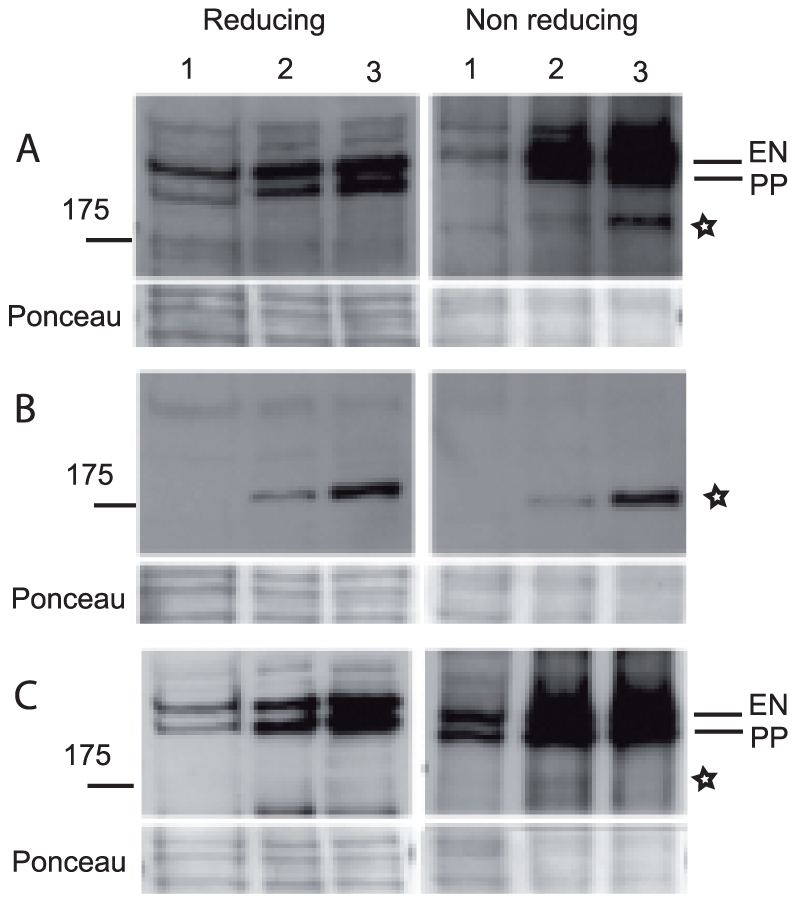
Denaturation conditions affect the recognition of p170 by PNP auto-antibodies by immunoblotting. Extracts (20 µg protein) from undifferentiated keratinocytes (lane 1), or differentiated for 4 days (lane 2) and 8 days (lane 3) were denatured in protein sample buffer containing or not 2-mercaptoethanol, separated on 8% SDS-PAGE and transferred onto nitrocellulose membrane for Western blot analysis using PNP serum 3 (A), anti-A2ML1 antibody (B), and PNP serum 2 (C). Sera numbering corresponds to that of Figure 1. Ponceau staining of the membranes is indicated as loading control. p170 migration level is indicated by a star. Envoplakin (EN) and periplakin (PP) migration levels are indicated.

So far, p170 has been exclusively detected by immunoprecipitation experiments, whereas immunoblotting studies using PNP sera invariably failed to detect p170 [7]. A2ML1 is a cysteine-rich protein with a predicted complex disulfide pattern [15], suggesting that A2ML1 possesses a very constrained structure. We therefore tested whether reducing conditions could play a role in the recognition of p170 by PNP sera by Western blotting. p170-positive PNP sera detected a protein of 170,000 from keratinocyte extracts under non-reducing conditions, but not reducing conditions (**Figure 2A**). The 170,000 protein was expressed more strongly by differentiated than by undifferentiated keratinocytes. This observation is in line with the reported increased expression of A2ML1 in the granular cell layers of the epidermis [15]. Interestingly, the reactive protein band showed the same electrophoretic migration of that recognized by the anti-A2ML1 antibody. The latter, however, bound to A2ML1 under both reducing and non-reducing conditions (**Figure 2B**). p170-negative PNP sera did not detect any 170,000 protein band under either reducing or non-reducing conditions (**Figure 2C**). These results suggest that PNP auto-antibodies recognize mainly conformation-dependent epitopes, which are lost under reducing denaturing conditions, explaining the lack of reactivity of PNP sera with p170 by Western blot analysis.

### Anti-A2ML1 antibodies recognize p170 immunoprecipitated by PNP sera

**Figure 3:**
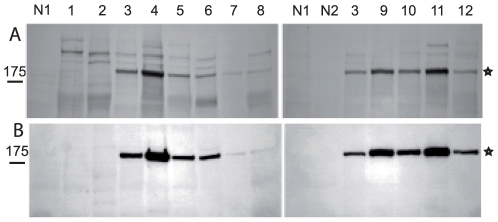
Anti-A2ML1 antibodies recognize p170 immunoprecipitated by PNP sera. Reduced samples immunoprecipitated from radiolabelled keratinocyte extracts with normal (N1, N2) or PNP sera (1 to 12) (see Fig. 1) were separated on 8% SDS-PAGE and transferred onto nitrocellulose membrane. This membrane was autoradiographed (A) and analyzed by immunoblotting using anti-A2ML1 antibodies (B).

The potential identity of p170 with A2ML1 was next analysed by immunoblotting of the radioactive PNP immunoprecipitates from keratinocyte extracts using anti-A2ML1 antibodies. A protein migrating exactly at the same level as S^35^-labelled p170 was recognized by the anti-A2ML1 antibody. A good correlation between the relative ratio of the signals obtained by Western blotting using the anti-A2ML1 antibodies and those of the autoradiogram (n = 10) was observed (**Figure 3**). In contrast, when immunoprecipitates obtained either with p170-negative PNP sera or normal sera were tested, no signal was detected using anti-A2ML1 antibodies (**Figure 3**). These results strongly suggest that p170 is A2ML1.

### A2ML1 expressed in transfected HEK 293T cells is recognized by p170-positive PNP sera

To further confirm the identity of p170 with A2ML1, we generated a cDNA construct for eukaryotic expression of a c-myc-tagged recombinant A2ML1 for both immunofluorescence and immunoprecipitation studies. First, we transfected Human Embryonic Kidney cells HEK 293T (American Type Culture Collection) and carried out double immunofluorescence microscopy studies using the PNP sera (serum 1 to 12) and an anti-c-myc antibody (representative sera are shown in **Figure S2**). The results showed that p170-positive PNP sera (serum 3 to 12) specifically labelled transfected cells expressing recombinant A2ML1-c-myc with a fine granular cytoplasmic staining, while p170-negative PNP sera (serum 1 and 2) and normal sera (serum N1 and N2) did not (**Figure S2**).

Western blot analysis showed that HEK 293T cells transfected with the construct encoding A2ML1-c-myc expressed and secreted a protein of Mr 170,000 as expected (**Figure S3**). The secretion of the c-myc-tagged A2ML1 in the culture medium was considered as an indicator for the proper folding of the protein.

**Figure 4:**
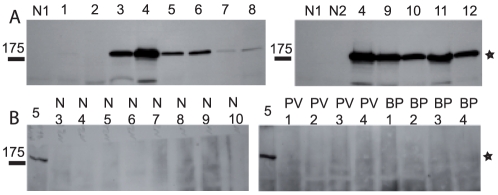
PNP sera immunoprecipitate recombinant A2ML1 expressed in HEK 293T. PNP (A, 1–12), normal (A and B, N1-N10), pemphigus vulgaris (PV1-PV4) and bullous pemphigoid sera (B, BP1-BP4) were used to immunoprecipitate proteins from extracts of HEK 293T cells transfected with pISb05, encoding recombinant A2ML1-c-myc. PNP sera numbering corresponds to that of Figure 1. Reduced immunoprecipitates were separated on 8% SDS-PAGE and analyzed by immunoblot using anti-c-myc antibody. p170 migration level is indicated by a star.

All p170-positive PNP sera (serum 3 to 12) immunoprecipitated A2ML1-c-myc from transfected HEK 293 cells, whereas p170-negative PNP sera (serum 1 and 2) did not (**Figure 4A**). The relative band intensities of the immunoprecipitates obtained from transfected cells using the different PNP sera paralleled those obtained using extracts from radiolabeled cultured keratinocytes. As control, sera from normal volunteers (n = 52), patients with pemphigus vulgaris (n = 24), pemphigus foliaceus (n = 4) and bullous pemphigoid (n = 28) did not immunoprecipitate A2ML1-c-myc from transfected HEK 293T cells (representative samples are presented **Figure 4B**). Together, these data provide strong support that p170 is A2ML1.

### Adsorption studies demonstrate that p170 corresponds to A2ML1

**Figure 5:**
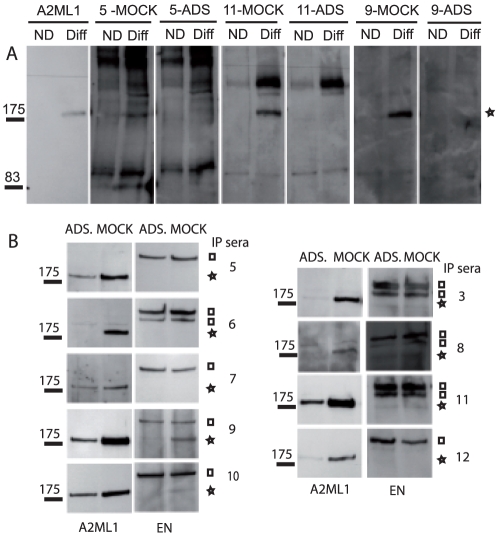
Adsorption of PNP sera with recombinant A2ML1 prevents PNP serum reactivity with p170. Nickel resin loaded with HEK 293T extracts expressing (ADS) or not (MOCK) A2ML1-Flag-HIS_8_ was incubated with PNP sera (diluted 1/10). Sera numbering corresponds to that of Figure 1. (A) Treated sera were used to carry out the Western blot analysis of undifferentiated (ND) or differentiated for 8 days (Diff) human primary keratinocyte extracts (20 µg/lane), separated after non-reducing denaturation on 6% SDS-PAGE. The migration position of p170 is marked by a star. (B) Treated sera were used to immunoprecipitate proteins from 8-day-differentiated primary human keratinocytes. Reduced immunoprecipitates were separated on 6% SDS-PAGE, and analyzed by Western blotting using anti-A2ML1 antibody (A2ML1). The same membrane was reprobed with anti-envoplakin (EN). The one or two bands corresponding to envoplakin (17) are marked by a square. The sequential probing of the membrane without stripping explains the signal at p170 level when probed with the anti-envoplakin antibody.

To provide additional evidence that A2ML1 corresponds to p170, we carried out immunoprecipitation studies using PNP sera that were adsorbed with recombinant A2ML1- FLAG-His_8_, expressed in transfected HEK 293T and bound to Ni^2+^-resin. As control, serum samples were treated with a Ni^2+^-resin loaded with mock-transfected cell extracts. To evaluate the selectivity of depletion, we first analyzed the pattern of immunoblotted proteins from undifferentiated and differentiated primary human keratinocyte extracts separated under non-reducing conditions (**Figure 5A**). Adsorbed PNP sera showed no or significantly reduced reactivity with p170, compared to mock-adsorbed PNP sera. Importantly, the binding of adsorbed sera to other PNP autoantigens was not affected, indicating that anti-p170 antibodies were selectively depleted by the A2ML1-FLAG-His_8_-affinity resin. We then carried out immunoprecipitation studies using differentiated primary human keratinocytes (**Figure 5B**). The immunoprecipitates obtained either with mock-adsorbed or adsorbed PNP sera (n = 9) were probed by Western blotting using the anti-A2ML1 antibody. Immunoprecipitation of p170 was either completely or at least strongly reduced when adsorbed sera were used, compared to mock-adsorbed sera. Immunoblotting of the same immunoprecipitates with anti-envoplakin antibodies [17] confirmed that the adsorption was selective for anti-A2ML1 antibodies in PNP sera. These results indicate that PNP autoantibodies bind to common epitopes present on both p170 and A2ML1.

Finally, we also assessed the effect of the depletion of the anti-p170 antibodies by immunofluorescence microscopy of skin cryosections using the PNP serum no. 9 with strong and apparently selective reactivity with p170 (**Figure S4**). The mock-adsorbed PNP serum stained the upper and superficial epidermal cell layers, the pattern of which was similar to that obtained with the anti-A2ML1 antibody (**Figure S5**), as previously described [15]. In contrast, there was almost no labelling of the upper epidermal layers when the adsorbed PNP serum sample was used.

### Mapping of A2ML1 domains recognized by PNP autoantibodies

**Figure 6:**
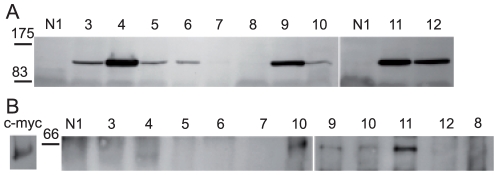
The NH_2_-half of A2ML1 is preferentially recognized by PNP antibodies. PNP sera (1–12) and normal serum (N1) (see Fig. 1) were used to immunoprecipitate recombinant A2ML1^1–889^-c-myc (A) or A2ML1^990–1454^-c-myc (B) expressed in transfected HEK293T cells. Immunoprecipitates were separated by SDS-PAGE after reducing (A) or non-reducing (B) denaturation to avoid the co-migration of the protein of interest with the antibodies used for the immunoprecipitation and analyzed by Western blotting using anti-c-myc antibody. As a control A2ML1^990–1454^-c-myc was immunoprecipitated in parallel with anti-c-myc antibodies (c-myc) and run together with the other samples. Exposure time of the c-myc lane was 2 sec versus 7 min for the others.

To gain insight about potential mechanisms by which auto-antibodies against A2ML1 may affect its function, we carried out a domain mapping study. A2ML1 was divided into two portions, based on the similarity between A2M and A2ML1 and A2ML1 predicted disulfide pattern [15], domains (http://smart.embl-heidelberg.de) and secondary structures (GOR4 program developed by NPS@: network Protein Sequence Analysis) [18], [19]. HEK 293T cells were transfected to express c-myc tagged recombinant forms of either the NH_2_-terminal half (A2ML1 ^1–889^) or the COOH-terminal half of A2ML1 (A2ML1 ^990–1454^). The former encompasses the bait domain important for protease targeting, whereas the latter contains the thiolester and low density lipoprotein receptor-related protein 1-binding domains [15], [20]. We assessed proper folding of these proteins by controlling their secretion (**Figure S3**). Nine out of ten PNP sera reacted with A2ML1 ^1–889^, while only three bound to the A2ML1 ^990–1454^, as revealed by immunoblotting of the immunoprecipitates with anti-myc antibodies (**Figure 6**). One serum did not show any reactivity. These results indicate that PNP autoantibodies mainly target the NH_2_-half of A2ML1.

## Discussion

The characterization of p170 antigen recognized by PNP sera has proved to be a challenging task. Our study unravels the identity of this protein targeted by PNP autoantibodies as the protease inhibitor A2ML1. This conclusion is based on several lines of evidence: 1) the tryptic mass profile of the immunoprecipitated p170 has a significant match with that expected for A2ML1; 2) p170 is recognized by anti-A2ML1 antibodies and can be immunoprecipitated from culture media of human keratinocytes, in the same manner as A2ML1; 3) PNP sera immunoprecipitate recombinant A2ML1 from cell extracts, whereas binding to A2ML1 was never observed with sera obtained from normal volunteers (n = 52) as well as patients with autoimmune bullous diseases of the skin (n = 56); 4) p170-reactive PNP sera selectively labelled transfected cells expressing recombinant A2ML1; 5) pre-incubation of p170-reactive PNP sera with recombinant A2ML1 selectively abrogated reactivity of the PNP sera with p170 by immunoblot or immunoprecipitation, and further reduced the labelling of the epidermal granular cell layers, where A2ML1 is predominantly expressed. Together, our data unravel a novel class of proteins targeted by autoantibodies in patients suffering from this devastating multiorgan disease.

A2ML1 is a broad range protease inhibitor belonging to the A2M protease inhibitor family. It binds different classes of proteases and inhibits their activity or reduces the substrate spectrum by a “trap mechanism” in which the inhibitor covalently binds the protease and creates a sterical hindrance within the active site [15]. During this process, the conformational change releases the COOH-terminal extension of A2ML1, which then can be recognized by lipoprotein receptor-related protein 1 (LRP1) receptor to allow the internalization and clearance of the complex protease inhibitor-protease [20]. A2ML1 is expressed in many tissues such as the epidermis, thymus, and testis, while A2ML1 ESTs reported in UniGene mainly arise from normal and tumoral stratified epithelia [15]. Furthermore, the EST expression profile (Hs.620532, NCBI Unigene, EST profile viewer) suggests the presence of high transcript levels of A2ML1 in oesophagus, mouth, pharynx, intestine, and muscle. Interestingly, comparison of the sequence of A8K2U0 and CAD48670, which displays the highest score with p170 tryptic mass profile suggests the existence of splice variant(s) of A2ML1.

The exact function of A2ML1 is not yet defined. By analogy to A2M which has been proposed to be an element of the innate immunity [21] A2ML1 may participate in defense mechanisms by binding to inflammatory cytokines, growth factors and by targeting a broad range of proteases [15]. Furthermore, A2ML1 is likely to be directly implicated in the maintenance of epidermal homeostasis based on its ability to form covalent complexes with the kallikrein KLK7 *in vitro* [15], a protease involved in proteolysis of intercellular structures and in desquamation process [22].

Our findings provide an explanation for the failure to identify p170 so far. First, the full length cDNA sequence of A2ML1 in humans was reported in 2004 [23], [24], while its functional characterization was carried out in 2006 [15]. Therefore, indexation and theoretical profiling of A2ML1 in databases have been only available in the past few years. Second, the biochemical properties of A2ML1 constitute an additional challenge for its identification. A2ML1 is a secreted glycoprotein with a secondary structure constrained by multiple disulfide bridges [15]. Furthermore, over-heating of A2ML1 in reducing-denaturing conditions results in its cleavage in two polypeptides of 120 kDa and 60 kDa ([15] and personal observations), which reduces the signal intensity at the expected electrophoretic migration. Finally, our analyses demonstrate that PNP anti-A2ML1 autoantibodies recognize conformational epitopes, explaining the two-decade failure to detect p170 by Western blot under denaturing conditions [7]. It is likely that production of A2ML1 in an eukaryotic expression system ensuring proper folding and posttranslational modifications facilitates its detection.

There is no ortholog of human A2ML1 in mouse, rendering the direct *in vivo* demonstration of the pathogenicity of anti-A2ML1 antibodies by passive transfer studies impossible. Accordingly, gene targeting experiments are not feasible. Furthermore, no hereditary human disorder has been mapped to the A2ML1 gene locus so far. Nevertheless, there are a number of indirect observations providing support to the idea that auto-antibodies against p170 are involved in the initiation or the perpetuation of tissue damage, since: 1) in a significant number of reported PNP patients, including patient 9 of the present study, autoantibody reactivity against p170 is found alone or with few additional reactivities, at least by immunoprecipitation [12], [13]; 2) PNP sera binding to p170 can be detected already at an early stage of the disease [12], [13]; 3) A2ML1 is not only expressed in skin but also in other organs affected in PNP (Hs.620532, NCBI Unigene, EST profile viewer); 4) acquired or genetic defects of protease inhibitors may cause a variety of muco-cutaneous diseases with systemic symptoms [22], [25], [26], [27]. For example, mutations in SPINK5 gene encoding the serine protease inhibitor lympho-epithelial Kazal-type inhibitor (LEKTI) cause Netherton syndrome [26] associated with chronic skin inflammation and skin barrier defects. LEKT1 is thought to regulate kallikrein activity [28], [29].

The impact of auto-antibodies on biochemical properties and function of A2ML1 could not be determined, due to difficulties to obtain sufficient amounts of purified A2ML1. However, two studies document the negative impact of the binding of autoantibodies to protease inhibitors. First, in acquired autoimmune angioedema, autoantibody binding to C1-inhibitor (C1-inh) facilitates its cleavage by its target proteases and result in a non-functional truncated circulating form of C1-inh [30]. Second, in rheumatoid arthritis, auto-antibodies to serpin E2 diminish the inhibitory activity of serpin on urokinase plasminogen activator serine protease [31]. In analogy, anti-A2ML1 antibodies may either destabilize A2ML1 or prevent its interaction with its target proteases, inflammatory cytokines or its receptor LRP1 and thereby affect the activity of extracellular proteases or amplify tissue damage. In this context, it should be noted that our domain mapping results show that the NH_2_-terminal portion of A2ML1 is almost systematically targeted by PNP autoantibody. Since this domain is implicated in the recognition of target proteases, PNP autoantibodies to A2ML1 may prevent the formation of protease-protease inhibitor complex. The identification of physiologically important targets of A2ML1 will be extremely useful to further understand the pathological involvement of anti-A2ML1 autoantibodies.

Dissecting the mechanisms underlying the association of PNP with distinct neoplasia is probably key for our understanding of the onset of autoimmunity in PNP. In the case of Castleman disease, tumor resection results in remission of PNP symptoms [2], [32], [33]. Castleman, thymoma or follicular dendritic cell sarcoma cells have been shown to produce autoantibodies reactive with various PNP autoantigens [33], [34]. Noteworthy, reactivity with p170 is found in up to 76% of patients with PNP associated with Castleman disease [32], [35]. Future systematic studies with prospective cohorts of patients and detailed analysis of the immunological profile are needed to assess whether presence of anti-A2ML1 autoantibodies is associated with a particular PNP phenotype and organ involvement as well as a specific type of neoplasia. In this context, the retrospective nature of our study precluded a reliable analysis. Nevertheless, based on the tissue distribution profile of A2ML1 and its lack of expression in pulmonary epithelium [36], it is unlikely that autoantibodies to A2ML1 contribute to bronchiolitis obliterans and respiratory failure, a frequent cause of death in PNP.

Our study thus puts an end to a relentless search for p170 and identifies PNP as a first example of an autoimmune multiorgan syndrom in which autoantibodies to a protease inhibitor might contribute to tissue damage by aggravating and precipitating inflammation.

## Materials and Methods

### Ethics Statement

Normal human serum samples were obtained from voluntary blood donors of the local regional Swiss blood bank. Written consent for public use was provided.

Patients' sera were obtained from patients managed in Switzerland (PNP n = 1, other bullous disease n = 56), France (PNP n = 12), Japan (PNP n = 7) and Germany (PNP n = 1). All sera were collected as a part of standard care and with the oral consent of the patients according to the local ethical rules. Sera were thus already available before this research was started. Since this is a non-interventional study and characterization of sera' reactivity is accepted and expected by the patients as normal diagnostic procedure, no written consent was required according to the local Swiss, French, German and Japanese ethics committees. Furthermore, since this research study did not involve intervention or interaction with the included individuals and the information collected during the study was not individually identifiable and not readily ascertained by the investigators, ethical review was not requested also in line with recent recommendations of the Office for Human Research Protections.

### Human sera

Sera were obtained from 20 patients with clinically, histologically and immunopathologically typical PNP [2], [3]. Sera were also obtained from patients with bullous pemphigoid (n = 28), pemphigus vulgaris (n = 24), pemphigus foliaceus (n = 4) and normal subjects (healthy blood donors, n = 52).

### Cell culture and transfection

Primary human keratinocytes (Invitrogen) were grown in Keratinocyte-SFM medium, supplemented with EGF and pituitary extracts (Invitrogen), penicillin, streptomycine. Differentiation was induced by adding calcium 1 mM, isoproterenol 1 µM and hydrocortisone 0.4 µg/ml to the growth medium [37]. Human Embryonic Kidney cells HEK 293T (American Type Culture Collection) cells were grown in DMEM supplemented with foetal bovine serum (FBS) 10%, penicillin, and streptomycine. HEK 293T cells were transfected according to the calcium phosphate method [38]. When HEK 293T cell culture medium was analyzed for protein secretion, the cells were briefly rinsed in PBS 15 hours after transfection and grown in DMEM medium without FBS for two days.

### In vivo labeling of keratinocytes

Primary human keratinocytes (foreskin) were purchased from Invitrogen. *In vivo* labelling of keratinocyte proteins was performed as previously described [10]. Briefly, after 4 days of differentiation, keratinocytes were incubated for 1 h in DMEM medium without methionin/cystein (Invitrogen) and then overnight in DMEM medium without methionin/cystein supplemented with 100 µCi/ml of ^35^S-Met/^35^S-Cys (Hartmann Analytics, Germany).

### Cell extracts

Cells were lyzed in Tris-HCl 50 mM pH 7.5, NaCl 150 mM, NP-40 1%, protease inhibitor cocktail (Sigma). The insoluble fraction was removed by centrifugation, and the supernatant was used for either immunoprecipitation or, after denaturation in protein sample buffer with or without 2-mercapto-ethanol, for separation on SDS-PAGE (6%) and Western blotting.

### Immunoprecipitation

Patient sera (30 µl) or anti-myc antibodies (4 µg) were incubated with protein A-Sepharose resin (GE Healthcare), in Tris-HCl 50 mM pH 7.5, NaCl 150 mM, protease inhibitor cocktail during 2 hours on ice. After discarding the unbound fraction, cell lysates were incubated with the resin 2 hours on ice, and then extensively washed with Tris-HCl 50 mM pH 7.5, NaCl 150 mM, protease inhibitor cocktail. Proteins bound to the resin were eluted in protein sample buffer (with or without 2-mercaptoethanol).

### In-gel digestion of p170 for MALDI mass spectrometry analysis

The preparative 1D-SDS-PAGE gel, used to separate the immunoprecipitated proteins, was stained with Coomassie Brilliant Blue R250. The band containing the 170,000 protein was excised, and transferred into a fresh quartz vessel. The gel pieces were washed alternately three times with 10 µl digestion buffer (10 mM NH_4_HCO_3_, pH 7.8) and 10 µl modified digestion buffer (10 mM NH4_H_CO_3_/acetonitrile 1∶1). Afterwards the gel pieces were shrunken in the vacuum and reswollen with 2 µl protease solution (0.05 µg/µl trypsin, Promega, USA). Digestion was performed for 10–12 h at 37°C. Afterwards 8 µl of 5% formic acid was added to the gel piece twice (successively) and the peptides were extracted for 15 min in a sonication bath. The pooled supernatant was mixed with 1 µl of C_18_-beads (Poros 10 R2- chromatography beads, 5 mg/ml in methanol, PerSeptive Biosystems, USA) and completely dried in a vacuum concentrator. Concentrated samples were taken in 1 µl MALDI matrix (α-cyano-hydroxycinnamic acid in acetonitrile/0.1% TFA, 0.7∶0.3).

### MALDI mass spectrometry and data analysis

MALDI peptide mass fingerprint analysis was done on a Reflex IV mass spectrometer (Bruker Daltonics,Germany). Matrix containing C_18_-bead bound peptides were transferred to a MALDI sample plate. Dried samples were washed with 1 µl of 0.1% TFA. Analysis was done with the following settings: a target voltage of 20 kV, an acceleration electrode voltage of 13.5 kV, a reflectron voltage of 21.6 kV and a reflectron detector voltage of 1.6 kV.

PMF spectra were interpreted using the Mascot program (http://matrixscience.com) [16]. The chosen parameters were: human NCBI and MSDB database, tryptic cleavage, mass tolerance ±1.5 Da, expected mass of the protein 170 kDa. The number of maximal missed cleavages was set to 1.

### Cloning

Clone DKFZp686O1010Q (AccNo AL832139) containing A2ML1 full length cDNA was purchased from ImaGenes (Germany). The vectors pISb05, pISb07, pISb08, pISb09 were derived from pcDNA3 vector (Invitrogen), and encode chimeric versions of A2ML1cDNA from clone DKFZp686O1010Q. pISb05 encodes A2ML1 fused to a c-myc-tag at its COOH-terminus, which was prepared in sequential steps. The c-myc-tag was introduced by PCR amplification using 5′GACTAGTCTCACAGGTCCTCCTCTGAGATCAGCTTCTGTTCTGCTTCACAGAGATCAGAATACTG and 5′CTTGCCAAATATGCCACTAC primers. A strong Kozak sequence was also inserted by PCR using 5′CCCAAGCTTGCCGCCACCATGTGGGCTCAGCTCCTTCTAG and 5′GAGAAGCCAAGGGAAACCTG primers. pISb07 is derived from pISb05, and encodes A2ML1 fused to a Flag-tag and a His_(8)_-tag at its C-terminus: this double tag was introduced by PCR using 5′ TGGTGAGTCAGGGTCTATGG and 5′TCCGGGCCCTCAATGATGATGATGATGATGATGATGACCGGTACGCTTGTCATCGTCATCCTTGTAGTCTGCTGCTTCACAGAGATCAGAATACTG primers. pISb08 encodes A2ML1 (residues 1–889) (A2ML1^1–889^) fused to a c-myc tag. This vector was derived from pISb05 and prepared using primers 5′ATGGGACAGACGTTTCTCTG and 5′ATAGGGCCCTCACAGGTCCTCCTCTGAGATCAGCTTCTGTTCTGCTGCGGGAACAAACCCCTTCTG to insert the c-myc tag sequence. pISb09 encodes A2ML1 (residues 890–1454) (A2ML1^890–1454^) fused to a C-myc tag, and was derived from pISb05. A strong Kozak sequence and a secretion signal peptide were introduced by PCR using 5′CCCAAGCTTGCCGCCACCATGTGGGCTCAGCTCCTTCTAGGAATGTTGGCCCTATCACCAGCCATTGCA CAAAAGGGCCGAAGTGACAC and 5′GTAGAGGTTGGTCGTGGAGG primers. The sequence of the portions of cDNA prepared and amplified by PCR were verified by sequencing (Mycrosynth, Switzerland).

### Preparation of protein from cell culture medium

Debris were removed from culture medium by centrifugation and proteins were precipitated with 10% v/v trichloroacetic acid 1 h on ice, the pellet was collected by centrifugation 10 min at 16,000 g, washed with acetone and resuspended in Laemmli sample buffer.

### Protein reducing and non reducing denaturation and separation

Protein reducing denaturation was performed in Laemmli buffer [39] incubated 3 min at 95°C degrees. For the non reducing denaturation, 2-mercaptoethanol was omitted. SDS-PAGE separation was carried out as previously described [39].

### Western blotting

Gels were transferred onto nitrocellulose membrane in Tris-glycine buffer, ethanol 10%, 0.1%SDS, for 1 h at 100 V. Western blot analyses were performed in standard conditions. Antibodies were diluted as follows: anti-A2ML1 1/250 (Abnova), anti-Flag 1/500 (Sigma-Aldrich), anti-c-myc 1/500 (Santa Cruz Biotechnology).

### Serum adsorption

Soluble extracts from HEK 293T cells transfected with A2ML1-FLAG-His_8_ were prepared as described above using a pH 8 buffer and were incubated with Nickel-resin (Sigma-Aldricht) 1 h on ice, and the resin was subsequently submitted to extensive washing with Tris-HCl 50 mM, pH 8, NaCl 200 mM, NP40 1%, protease inhibitor. The equilibrated resin was incubated overnight with 100 µl serum diluted 10 fold in the same buffer with protease inhibitors. The same procedure was performed using untransfected HEK 293T cell extracts for the control experiment. The supernatant was then used for Western blot analysis, immunoprecipitation or immuno-fluorescence.

### Immunofluorescence

All antibodies were diluted in PBS 1% BSA. HEK 293T cells were fixed with paraformaldehyde 4%, permeabilized with Triton X-100 0.1%, blocked with BSA 1%, and incubated with Anti-c-myc (1/150) and patient sera (1/150) during 1 h. The Alexa-fluor 488-anti-rabbit and Alexa-fluor-588-anti-human secondary antibody (Invitrogen) were diluted 1/300 and incubated 1 h at room temperature. Nuclei were stained with DAPI. Cryostat sections (4–5 µm) of human breast skin were air-dried, fixed for 10 min in 4% ice cold acetone and rehydrated in Tris–buffered saline with 0.1% saponin. Double immunofluorescence was performed by serially incubating sections with anti-A2ML1 (1/50) and anti-envoplakin CR5 (1/100) antibody for 1 h followed by incubation with Alexa-fluor-568-anti-mouse (1/100) and Alexa-fluor-588-anti-rabbit (1/100). Nuclei were stained with DAPI. Immunofluorescence with adsorbed sera was performed incubating the adsorbed and mock-adsorbed sera (1/2.5) 1 h, and then Alexa-fluor-568 anti-human antibody (Invitrogen).

## Supporting Information

Figure S1 Monoisotopic mass profiles of p170 tryptic digest (MALDI-TOF mass spectrometry).
(9.64 MB EPS)

Figure S2 PNP sera selectively recognize HEK 293T cells expressing recombinant A2ML1-c-myc. Cells transfected with pISb05, encoding A2ML1-c-myc were labelled with both anti-c-myc antibody (green) and human sera (red): normal (A) or PNP 2, 4 and 7 (B, C and D). Nuclei were stained with DAPI (blue). Sera numbering corresponds to that of Figure 1. White scale bar: 100 µm.
(2.14 MB EPS)

Figure S3 Expression and secretion of recombinant A2ML1 proteins in HEK 293T cells. Extracts (20 µg) of untransfected HEK 293T cells (lanes 1) or transfected with pISb07 (lanes 2), encoding A2ML1-Flag-His8, were separated on 10% SDS-PAGE, and analyzed by Western blotting using anti-A2ML1 or anti-Flag antibody. B. Culture medium (400 µl) of 293T cells non transfected (lane 1), or transfected with pISb05 (lane 2), pISb08 (lane 3), pISb09 (lane 4) encoding A2ML1-c-myc, A2ML11-889-c-myc, and A2ML1890-1454-c-myc, respectively were TCA-precipitated, separated on 10% SDS-PAGE and analyzed by Western blotting using anti-c-myc antibody. Lane 2, the band at 60,000 is likely a COOH-terminal degradation product of A2ML1 occurring during denaturation of the sample under reducing conditions.
(1.95 MB EPS)

Figure S4 Adsorption of PNP-serum 9 with recombinant A2ML1 reduces immunofluorescence staining in the upper layers of epidermis. PNP serum 9 mock-adsorbed (A) or adsorbed with recombinant A2ML1 (B) were used to probe sections of human breast skin. The reactivity of the secondary anti-human antibodies against human breast skin section is shown (C): the signal detected in the dermis and the upper cornified edge of the epidermis is therefore non-specific to the patient serum reactivity. The specific reactivity due to A2ML1 is expected in the upper layers of the epidermis, areas indicated by the white arrows. As a reference A2ML1 pattern in human breast skin is shown. Nuclei were stained with DAPI (blue). White scale bar: 100 µm.
(2.19 MB EPS)

Figure S5 A2ML1 is expressed in the upper layers of human skin. Sections of human breast skin were labeled with CR5 anti-envoplakin antibody (A, green) and anti-A2ML1 antibody (B, red). The patterns obtained with the secondary anti-rabbit (C, green) and anti-mouse antibody (D, red) are shown. Nuclei were stained with DAPI. White scale bar: 100 µm.
(1.74 MB EPS)
